# DIDS (4,4'-Diisothiocyanatostilbene-2,2'-disulfonate) directly inhibits caspase activity in HeLa cell lysates

**DOI:** 10.1038/cddiscovery.2015.37

**Published:** 2015-09-28

**Authors:** E Benítez-Rangel, MC López-Méndez, L García, A Guerrero-Hernández

**Affiliations:** 1 Department of Biochemistry CINVESTAV-IPN, Apdo. Postal 14-750, Mexico, D.F. México

## Abstract

Apoptosis is an important mechanism of cell demise in multicellular organisms and Cl^−^ transport has an important role in the progression of the apoptotic volume decrease (AVD). DIDS (4,4'-Diisothiocyanatostilbene-2,2'-disulfonate) is one of the most commonly used Cl^−^ transport inhibitors that eliminates or reduces different apoptotic hallmarks such as AVD, caspase-3 activity and DNA fragmentation. DIDS is also a protein crosslinker that alkylates either amino or thiol groups. Since caspases are thiol proteases, our aim was to study whether DIDS could directly inhibit the activity of these proteases. Here, we show that caspase activity induced by 4 h incubation with staurosporine was inhibited by DIDS in HeLa cells that were maintained in the absence of serum for 24 h. Interestingly, the caspase-inhibitory effect of DIDS is downstream to the inhibition of cytochrome c release, suggesting that DIDS might be also acting at the apoptosome. Moreover, DIDS was able to inhibit capase-3, -9, and -8 activities in cell lysates, implying that DIDS can react with and directly block caspases. Our data suggest that antiapoptotic activity of DIDS involves not only inhibition of the voltage-dependent anion channel (VDAC) at the mitochondria and Cl^−^ channels at the plasma membrane, but also a third mechanism based on the direct inhibition of caspases.

## Introduction

Apoptosis, one form of programmed cell death, is an important mechanism of cell demise in multicellular organisms. It has been established that ion fluxes, particularly K^+^ efflux, are required for the apoptotic process. The early phase of apoptotic cell shrinkage is characterized by alterations in the activity and regulation of membrane ion channels.^1–4^ Cl^−^ transport activation is also required during apoptosis,^3^ mainly in the progression of the apoptotic volume decrease (AVD).^5^ It has been suggested that Cl^−^ channels and/or Cl^−^ exchangers, such as Cl^−^/ HCO_3_^−^, have an apoptotic role in different cell types including HeLa cells,^6^ cerebellar granule neurons,^7^ salmonid cells,^8^ cardiomiocytes,^9,10^ renal proximal tubule cells,^11^ thymocytes^12^ and HL60 cells.^13^ This activation of Cl^−^ transport occurs in response to different apoptosis inducers such as staurosporine (STS), tumor necrosis factor (TNF), cycloheximide,^5,14^ etoposide, H_2_O_2_ or Fas.^14^ One widely used substance to assess the participation of Cl^−^ transport in apoptosis is DIDS (4,4'-Diisothiocyanatostilbene-2,2'-disulfonate), which is able to inhibit AVD triggered by different apoptotic inducers,^5^ and dramatically diminishes the number of apoptotic cells.^7,9,11^ DIDS has also been shown to block other hallmarks of apoptosis such as caspase-3 activity^7,9,11,14^ and DNA fragmentation.^9^

It has been demonstrated that inhibition of the Cl^−^/HCO_3_^−^ exchanger by DIDS depends on two main characteristics of this molecule; namely, being an anion due to the presence of sulfonate and being an alkylating agent of amino groups due to the presence of isothiocyanate residues. Accordingly, the lysines alkylated by DIDS in the Cl^−^/HCO_3_^−^ exchanger have been identified.^15^ DIDS inhibits apoptosis by targeting anion transporters at two different cell locations, the plasma membrane and the outer mitochondrial membrane. The former reflects Cl^−^ channels and Cl^−^/HCO_3_^−^ exchangers that are sensitive to DIDS and are involved in AVD,^5^ while the latter encompasses VDAC that participates in apoptosis as one of the pathways for releasing cytochrome c to the cytoplasm.^16^ The ability of DIDS to inhibit VDAC in intact cells^17–19^ implies that this inhibitor is able to reach the cell interior when it should not; because of its hydrophilic nature due to the presence of sulfonate groups. It is well known that DIDS is able to alkylate amino groups of lysine residues,^20^ but DIDS can also alkylate thiol residues in proteins. Indeed, it has been demonstrated that DIDS inhibits the activity of the plasma membrane Ca^2+^ ATPase (PMCA) by alkylating thiol groups of this protein.^21^ Moreover, DIDS induces the mitochondrial permeability transition pore by its thiol crosslinking activity.^22,23^ Since caspases are thiol proteases, we studied whether DIDS had any inhibitory effect on these proteases. We describe here a third mechanism for DIDS to inhibit apoptosis, which involves the direct inhibition of caspase activity. All these different targets of DIDS might explain its generalized antiapoptotic activity seen in different cell types. Importantly, our data show that 50 *μ*M DIDS is enough to inhibit apoptosis and that a higher concentration of DIDS (500 *μ*M) induced a new DEVDase activity (unrelated to caspase-3) that may explain the deleterious cellular effect that can also be displayed by DIDS.

## Results

### DIDS that has been irreversibly bound to HeLa cells reduced both [Ca^2+^]_i_ and plasma membrane potential responses to histamine

**Figure 1:**
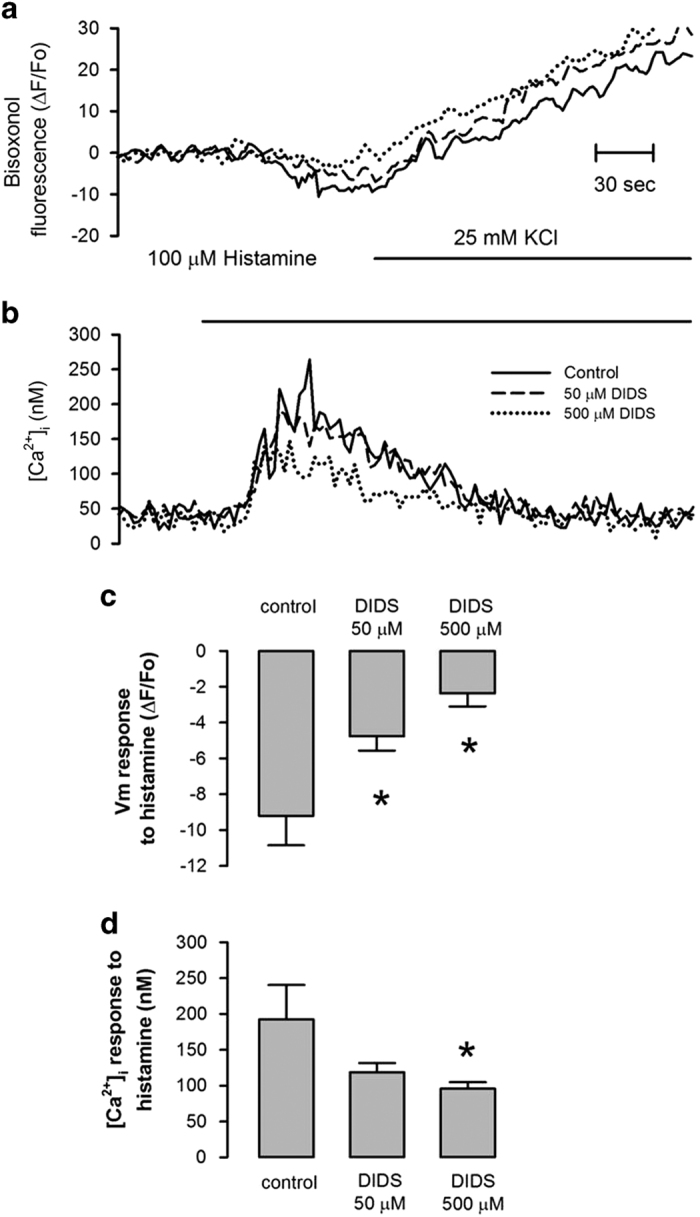
DIDS irreversibly alters histamine-induced PMP and [Ca^**2+**^]_i_ responses in HeLa cells. Simultaneous recordings of the effect of DIDS preincubation on plasma membrane potential (PMP) (**a**) and [Ca^2+^]_i_ (**b**) in response to histamine and elevation of external [K^+^] (applied where indicated). Representative recordings in DIDS-free saline solution of cells that had been preincubated without (solid line) or with DIDS 50 (dashed line) or 500 *μ*M (dotted line) for 2 h. Note that histamine induced membrane hyperpolarization (**a**), as a result of the activation of Ca^2+^-dependent K^+^ channels by the histamine-induced [Ca^2+^]_i_ response (**b**). Preincubation of cells with either 50 or 500 *μ*M DIDS decreased histamine-induced PMP response (**c**) while only 500 *μ*M DIDS reduced histamine-induced [Ca^2+^]_i_ response (**d**). (*n*=3, *P<0.05).

To determine whether DIDS affected HeLa cells [Ca^2+^]_i_ responses, we assessed both [Ca^2+^]_i_ and plasma membrane potential in response to activation of H1 receptors by histamine. To this end, HeLa cells were preincubated with either 50 or 500 *μ*M DIDS for 2 h and washed to remove unbound DIDS. Preincubation with DIDS inhibited histamine-induced hyperpolarization of the PMP from −9.2±1.6% ΔF/Fo (*n*=3) in the absence of DIDS to −4.8±0.79% ΔF/Fo (*n*=3) and −2.4±0.7% ΔF/Fo (*n*=3) for 50 and 500 *μ*M DIDS, respectively (Figures 1a and c). It is known that the histamine-induced hyperpolarization involves the activation of Ca^2+^-dependent K^+^ channels, by IP_3_-mediated Ca^2+^ release from internal stores.^24^ Histamine-induced [Ca^2+^]_i_ responses were reduced from 192.5±47.8 nM (*n*=3) in the absence of DIDS to 118.4±13.1 nM (*n*=3) and 95.7±9.1 nM (*n*=3) when cells were preincubated with 50 and 500 *μ*M DIDS, respectively (Figures 1b and d). DIDS did not have any direct effect on Bisoxonol fluorescence (Supplementary Figure S1); however, this was not the case for Fura-2 fluorescence which was quenched by DIDS addition to cells loaded with Fura-2 (Supplementary Figure S2). For this reason cells were preincubated with DIDS and thoroughly washed to eliminate unbound DIDS before loading these cells with Fura-2 (Supplementary Figure S1). Therefore, this inhibitory effect of DIDS on histamine-induced [Ca^2+^]_i_ response in washed cells, that was recorded in the absence of DIDS, suggests the irreversible alkylation of signaling proteins by DIDS.

### DIDS inhibits the activation of caspases induced by staurosporine

**Figure 2:**
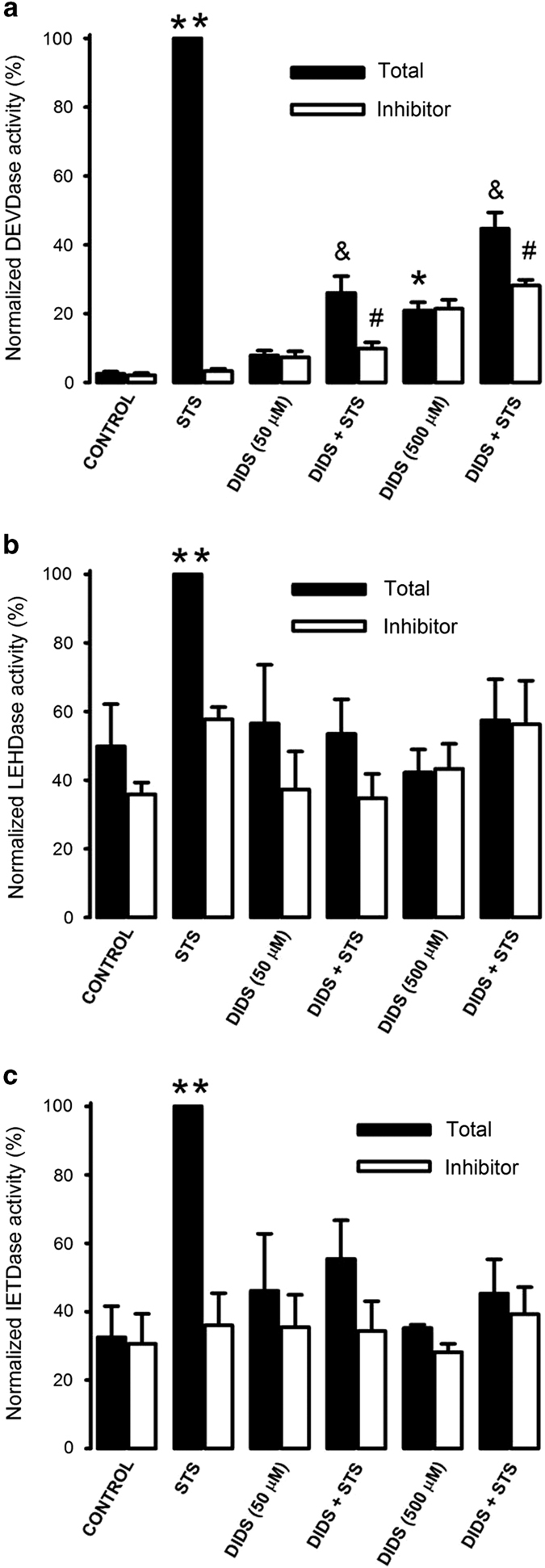
DIDS inhibits STS-induced caspase activation in HeLa cells. Cells that were preincubated with or without DIDS (50 or 500 *μ*M) for 30 min were incubated with STS 1 *μ*M for 4 h and lysed to determine caspase activity, which was normalized based on the response obtained with STS. Panels show caspase-3 (**a**, *n*=7), capase-9 (**b**, *n*=4) and caspase-8 (**c**, *n*=4) activities without (total activity, solid columns) and with caspase inhibitors (open columns), which were Ac-DEVD-CHO for caspase-3 (**a**), Ac-LEHD-CHO for caspase-9 (**b**) and Ac-IETD-CHO for capase-8 (**c**).ln all cases, STS induced a significant caspase activity with respect to control (**P<0.05). *^,&,#^*P*<0.05, Tukey multiple comparison test. *DIDS 500 *μ*M increased DEVDase activity unrelated to caspase-3 when compared with control activity. ^&^STS-induced caspase-3 activity is significantly smaller in the presence of either 50 or 500 *μ*M DIDS. ^#^There is a small but significant caspase-3 activity resistant to DIDS.

It has been shown that chloride flux^5^ and VDAC^17,25^ participate in apoptosis, since their inhibition with DIDS, among other compounds, reduces apoptosis. Indeed, preincubation of HeLa cells with DIDS for 30 min inhibited STS-induced activation of caspases-3, -9 and -8 (Figure 2). For all the caspases assessed, the low concentration of DIDS (50 *μ*M) displayed the maximal inhibitory effect on STS-induced activation of caspases. However, there was a small fraction of caspase-3 activity (approximately 15%) that was equally resistant to both 50 and 500 *μ*M DIDS (Figure 2a). This was not the case for caspase-9 and caspase-8 since they were fully blocked by 50 *μ*M DIDS (Figures 2b and c). Unexpectedly, DIDS by itself induced DEVDase activity resistant to the inhibitor of caspase-3 (Ac-DEVD-CHO), increasing by 3-fold with 50 *μ*M and by 8-fold with 500 *μ*M. The latter was large enough to be significant (Figure 2a). However, this new effect of DIDS was specific for DEVDase since it increased neither LEHDase (caspase-9, Figure 2b) nor IETDase (caspase-8, Figure 2c) activities. It is important to highlight that this DEVDase activity resistant to the caspase-3 inhibitor was only induced by DIDS; because other ion channel inhibitors like flufenamic acid or 2-APB (that inhibit apoptosis; Supplementary Figure S3) did not increase DEVDase activity. These data suggest that DIDS (50 *μ*M) inhibits activation of both initiator and execution caspases and that DIDS at high concentrations (500 *μ*M) induced a new DEVDase activity that was resistant to the caspase-3 inhibitor.

### DIDS did not inhibit STS-induced cytochrome c release

**Figure 3:**
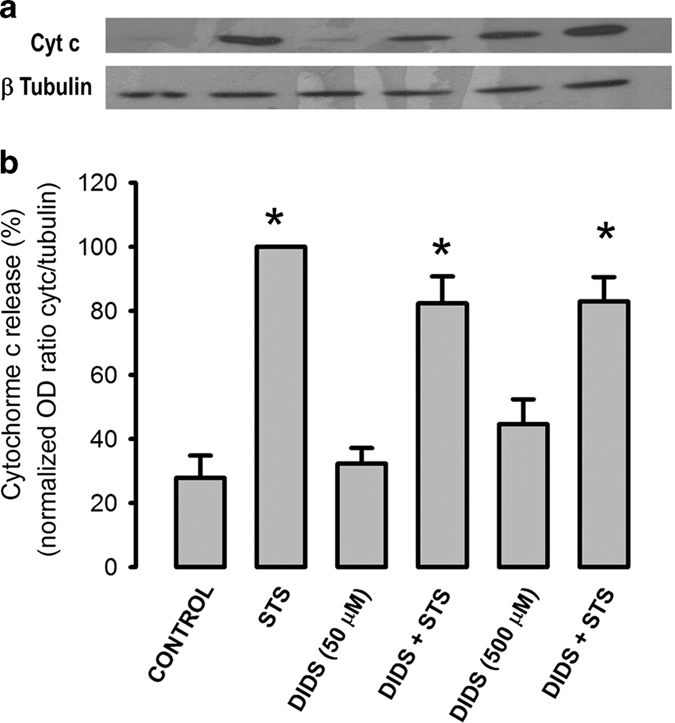
DIDS does not abolish STS-induced cyt c release. HeLa cells that were in the absence of serum for 19.5 h were preincubated with or without DIDS (50 or 500 *μ*M) for 30 min followed by STS (1 *μ*M) incubation for 4 h and lysed to determine cytochrome c release from mitochondria. (**a**) Representative western blots of cytochrome c (cyt c) and p tubulin in supernatants of cell lysates. Note that DIDS 500 *μ*M release some cyt c by itself. (**b**) The optical density ratio between signals of cyt c and p-tubulin was normalized using the STS-induced ratio as 100%. DIDS did not inhibit cytochrome c release, only reduced it by 20% at both concentrations of DIDS (*n*=7). *P<0.05 between staurosporine-induced ratio and the corresponding control condition.

The inhibitory effect of DIDS on caspase-9 activity might suggest that this agent affects apoptosis by blocking upstream events of caspase activation. In this regard, we studied the effect of DIDS on STS-induced cytochrome c release in HeLa cells that had been without serum for 24 h and used tubulin as a loading control (Figure 3a). To this end, cells were lysed and the supernatant separated by centrifugation at 10 000×*g* for western blot. The protein content of these fractions was not affected by incubation with STS (Supplementary Figure S4). Neither 50 nor 500 *μ*M DIDS significantly reduced STS-induced cytochrome c release that was normalized against tubulin, if anything there was only a slight reduction (Figure 3b, *n*=7). Interestingly, high concentrations of DIDS (500 *μ*M) alone induced to some extent release of cytochrome c (Figure 3). We have determined that the effect of STS on cytochrome c release was not blocked by either a pan-caspase inhibitor^26^ or 1 *μ*M cyclosporine A (not shown), the latter to inhibit activation of the mitochondrial permeability transition pore. However, we have shown that high external [K^+^] or the combination of K^+^ channels blockers such as TEA^+^ and 4AP significantly inhibited STS-induced cytochrome c release.^26^ These data suggest that DIDS inhibits caspase activation downstream of cytochrome c release in STS-induced apoptosis of HeLa cells.

### DIDS directly inhibits caspase activity in cell lysates

**Figure 4:**
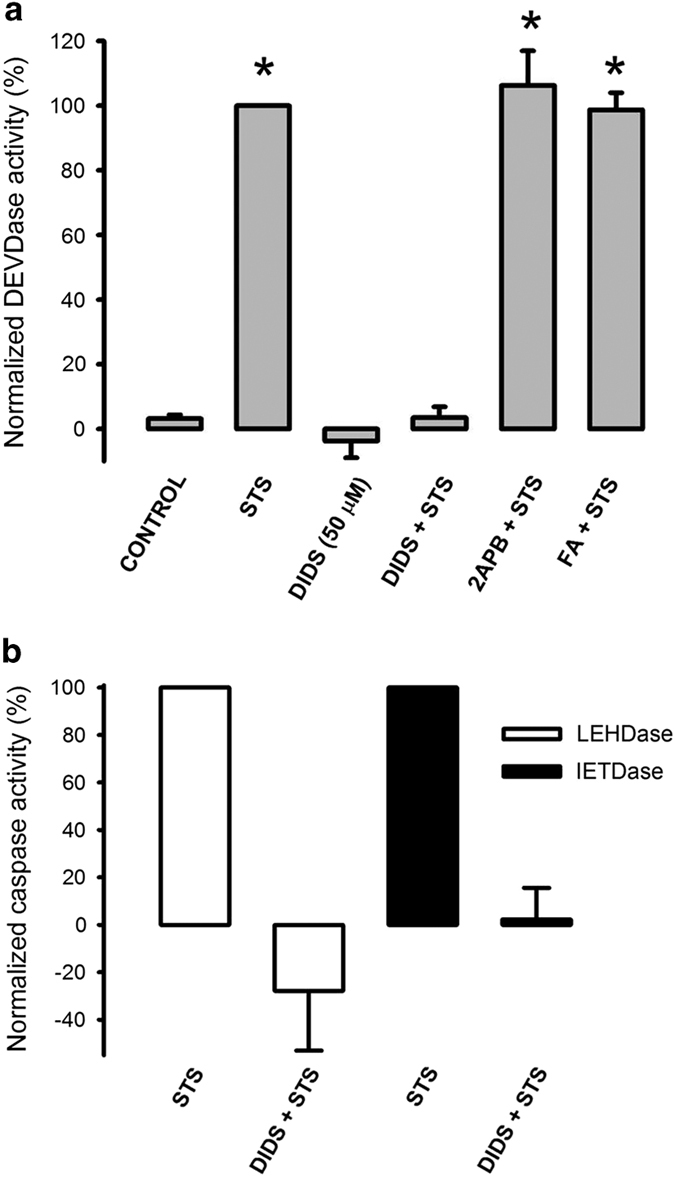
DIDS totally eliminates caspase activities in a cell-free extract. HeLa cells that were previously treated with or without STS (1 *μ*M) for 4 h were lysed and these extracts were incubated with DIDS (50 nM), 2-APB (50 nM) or flufenamic acid (50 nM) for 45 min at 37 °C followed by the corresponding caspase activity procedure. Note that DIDS but not the other ion channel inhibitors abolished caspase-3 (**a**, *n*=5), caspase-9 (LEHDase) and caspase-8 (lETDase) activities (**b**, *n*=3). Neither 2-APB nor flufenamic acid induced any DEVDase activity (not shown). *P<0.05 when compared with respective controls.

Because DIDS did not inhibit STS-induced cytochrome c release, yet fully blocked activation of caspases, particularly caspase-9; we decided to study whether DIDS had any direct inhibitory effect on caspases. To this end, we incubated cells with STS for 4 h and prepared cell lysates that contain fully activated caspases. These lysates were further incubated with 50 *μ*M DIDS for 45 min at 32 °C and the remaining caspase activities were assessed. In all cases, this procedure led to complete inhibition of previously activated caspases-3, -9 and -8 (Figure 4). Incubation of these cell extracts with other ion channel blockers, for example, 2-APB (50 *μ*M) a non-specific inhibitor of ion channels including; IP_3_R,^27^ Orai^28^ and TRPM2 channel^29^ or flufenamic acid (50 *μ*M) an inhibitor of Cl^−^ and cation channels,^30^ did not have any effect on the previously activated caspase-3 (Figure 4a). Collectively, these data imply that DIDS can directly inhibit the activity of caspases by a mechanism that is unrelated to the blocking effect of this substance on the activity of Cl^−^ transport and VDAC.

### DIDS inhibits STS-induced procaspase-3 processing, caspase-3 substrate degradation and apoptotic nuclei

**Figure 5:**
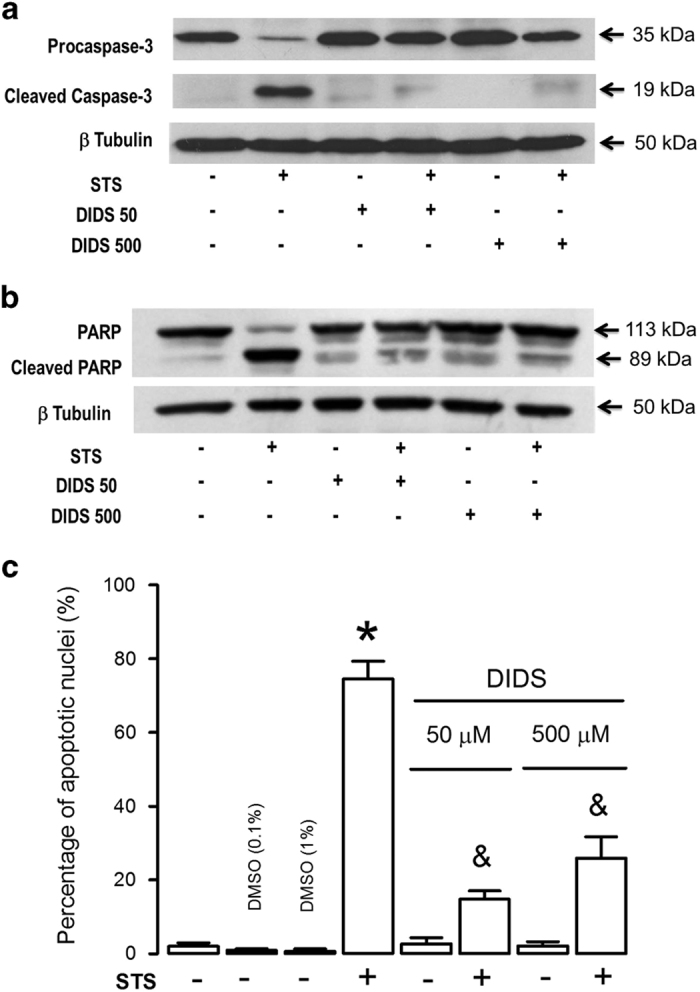
DIDS inhibits caspase-3 processing, PARP degradation and apoptotic nuclei features. HeLa cells preincubated with or without DIDS (50 or 500 *μ*M) for 30 min before incubation with STS (1 *μ*M) for 4 h were lysed and (**a**) pro-caspase 3 was detected with antibody (clone 3G2, *n*=3) and (**b**) PARP fragmentation with antibody (clone 4C10-5, *n*=5). DIDS totally abolished procaspase-3 proteolytic processing and strongly diminished STS-induced PARP fragmentation. (**c**) Apoptotic nuclei were induced by STS (4 h incubation). However, neither the vehicle (DMSO, 0.1 or 1%) nor DIDS alone induced apoptotic nuclei. Preincubation with DIDS (50 *μ*M) strongly inhibited STS-induced apoptotic nuclei features. Interestingly, this inhibitory effect was a little bit smaller for a 10 times higher concentration of DIDS (500 *μ*M) *^,&^*P*<0.05, Tukey multiple comparison test. *respect the control, ^&^respect the effect of STS.

To verify that DIDS has an inhibitory effect on initiator caspases and caspase-3, we studied the effect of DIDS on the processing of procaspase-3 and the cleavage of a known substrate of caspase-3 by western blot assays. As expected, STS produced a complete processing of procaspase-3 (35 kDa), a situation that was strongly inhibited by preincubating the cells with either 50 or 500 *μ*M DIDS (Figure 5a). Accordingly, STS was able to generate a cleaved form of caspase-3 (17–19 kDa). The amount of this fragment was strongly diminished by the preincubation with DIDS (Figure 5a, *n*=3). PARP enzyme has been shown to be one of the substrates of caspase-3,^31^ as expected STS induced cleavage of PARP from the original 113 kDa to a smaller fragment of 89 kDa. This effect was dramatically reduced by preincubation of cells with either 50 or 500 *μ*M DIDS (Figure 5b, *n*=5). Additionally, we have studied the effect of STS alone and in combination with DIDS on the nuclear morphology using confocal microscopy (Supplementary Figure S5). Control cells, which had been in the absence of serum for 24 h, showed normal nuclear morphology that was not altered by either DMSO vehicle (0.1 or 1%) or DIDS alone (50 or 500 *μ*M). However, STS (1 *μ*M for 4 h) induced on average 75% of apoptotic nuclei. DIDS (50 *μ*M) inhibited this effect since only 15% nuclei showed apoptotic features. Increasing the DIDS concentration (500 *μ*M) did not result in higher inhibition, if anything, a slightly decreased effect was now observed with 25% of the nuclei showing apoptotic characteristics (Figure 5c). Collectively, these data suggest that DIDS (50 *μ*M) inhibits STS-induced procaspase-3 processing, PARP degradation and apoptotic nuclear features. Increasing the DIDS concentrations (500 *μ*M) did not augment any of these parameters. Actually, this higher concentration showed signs of some deleterious effects.

### DIDS permeability in HeLa cells

**Figure 6:**
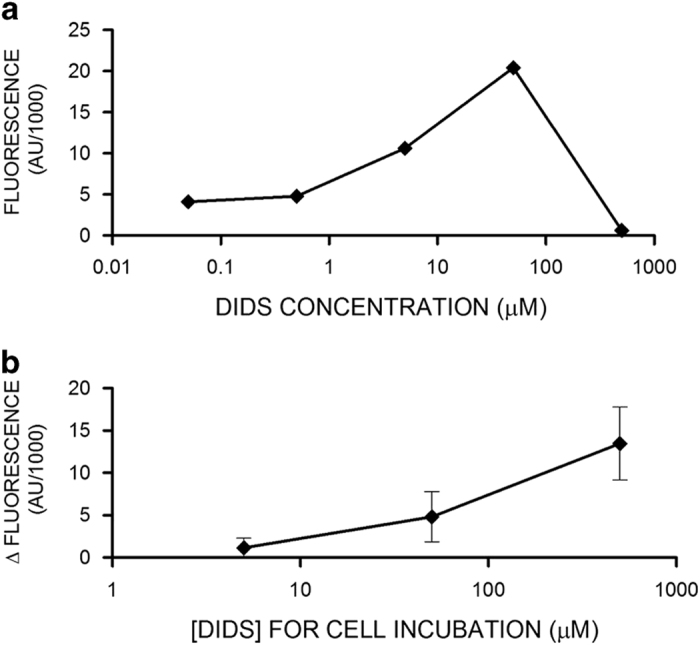
DIDS binds irreversibly to HeLa cells and make them fluorescent. (**a**) DIDS fluorescence signal as a function of concentration shows a biphasic behavior. DIDS concentrations higher than 50 *μ*M displayed a reduction in fluorescence signal most likely due to internal quenching. (**b**) HeLa cells preincubated with DIDS (5, 50 or 500 *μ*M) for 2 h were washed and fluorescence due to DIDS (420 nm) was determined (*n*=3). HeLa cells autofluorescence was substracted from the signal obtained for those preincubated with DIDS. Collectively, these data suggest that preincubating cells with 50 *μ*M DIDS leads to an accumulation of this inhibitor of at least 5 *μ*M inside cells.

**Figure 7:**
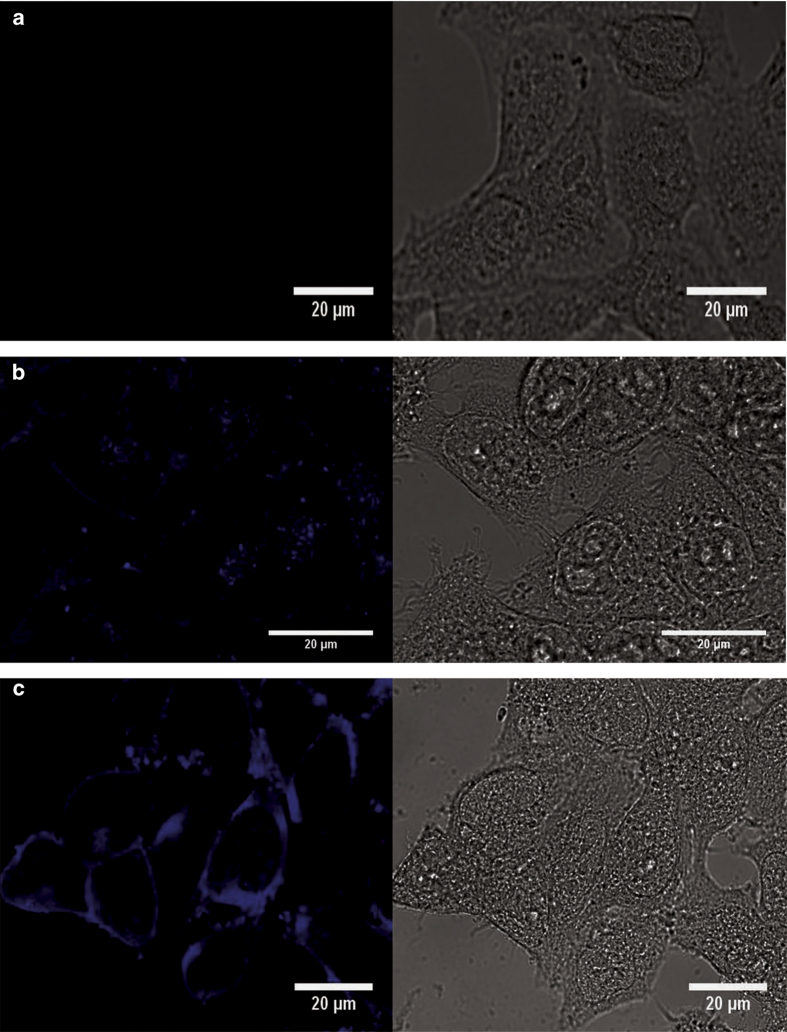
Confocal imaging of DIDS-labeled HeLa cells. (**a**) Control cells, because were not incubated with DIDS, display no fluorescence signal. (**b**) HeLa cells that were incubated with 50 *μ*M DIDS showed labeling of cell periphery and a vesicular structure that is located in close proximity to the nucleus. (**c**) Higher concentrations of DIDS (500 *μ*M) produced a fluorescent signal of larger intensity particularly at the cell periphery but also inside cells. Importantly, bright field image of HeLa cells with 500 *μ*M DIDS do not look as healthy as control cells. These cells show some donut-shaped structures that are stained by DIDS.

Our previous data suggest that DIDS binds to proteins in an irreversible manner as it has been shown in other systems.^15^ To corroborate this situation, we took advantage of the fluorescent properties of DIDS.^32^ We carried out fluorescence spectra at different concentrations of DIDS, exciting at 345 nm wavelength and detecting fluorescence at 420 nm wavelength, we observed a concentration-dependent increase in DIDS fluorescence up to 50 *μ*M; at 500 *μ*M DIDS fluorescence decreased most likely by a process known as internal quenching (Figure 6a), making it difficult to assess the concentration at high levels with the use of fluorescence; however, fluorescence can be used to estimate concentrations lower than 50 *μ*M DIDS under our recording conditions. Accordingly, HeLa cells were preincubated with 5, 50 and 500 *μ*M DIDS for at least 1 h and then thoroughly washed before recording their fluorescence. DIDS at 5 *μ*M did not significantly increase HeLa cell fluorescence; however, 50 and 500 *μ*M DIDS increased cell fluorescence (Figure 6b), the former increased HeLa cell fluorescence to levels comparable to an aqueous solution of 5 *μ*M DIDS. Since we used only 300 000 cells per ml of recording solution, we think it is unlikely that all the DIDS is packed in the plasma membrane, mainly because we expect that this packaging of DIDS at the plasma membrane would quench DIDS fluorescence. It seems feasible that a large fraction of DIDS is internalized and bound to cell proteins arguing for the idea that under our incubation conditions (37 °C for 4.5 h) DIDS has been incorporated into HeLa cells reaching internal proteins. To verify this conclusion, we have carried out confocal microscopy studies of DIDS cellular localization. Control cells did not show any fluorescent signal excited at 405 nm (Figure 7a). Cells incubated with DIDS (50 *μ*M) for 2 h displayed a stained plasma membrane and also a vesicular structure located close to the nucleus (Figure 7b). Cells in 500 *μ*M DIDS displayed a higher fluorescence signal both at the periphery and inside cells (Figure 7c). These data suggest that DIDS binds to cells and internalizes in an irreversible manner because these cells were thoroughly washed to remove unbound DIDS before fixation.

## Discussion

We have found in this study that DIDS was unable to inhibit cytochrome c release in HeLa cells that had been in the absence of serum for 24 h and exposed to STS for only 4 h. These data suggest that there is another target for DIDS downstream of the mitochondrial stage of apoptosis, and that this new target is not an anion transporter. We have demonstrated that DIDS at 50 *μ*M, totally and directly inhibits previously activated caspases-3, -9 and -8 in cell lysates. However, under our experimental conditions, the effect appears to be more complex, since in cells, there is a small fraction of caspase-3 that was resistant to DIDS and high concentrations of DIDS (500 *μ*M) induced a DEVDase activity that cannot be ascribed to caspase 3 because it was not inhibited by its corresponding inhibitor. Interestingly, it has been reported that DIDS (500 *μ*M) fully inhibits STS-induced apoptosis, while having no effect on etoposide- or TNF-induced cell death in Jurkat cells.^14^

AVD appears to have an important role in some forms of apoptosis,^5^ because the reduction of intracellular concentrations of both K^+^ and Cl^−^ ions, via different types of ion channels or transporters, is required for the activation of caspases. In this regard, it has been shown that different types of inhibitors of either K^+^ or Cl^−^ flux inhibit apoptosis to some extent.^2,4^ Accordingly, DIDS has frequently been used as an inhibitor of either Cl^−^ channels^3^ or Cl^−^/HCO_3_^−^ exchanger,^15^ and inhibits AVD produced by different and unrelated apoptosis inducers such as STS, TNF, etoposide, dexametasone, serum removal and hypoxia.^5,14^ This effect of DIDS on AVD is also shared by many other inhibitors of Cl^−^ transport such as NPPB and SITS.^5^ All these inhibitors also reduced cell death regardless of the inducer used.^5^ Interestingly, there are some cases where DIDS blocks apoptosis more strongly than the other Cl^−^ transport inhibitors such as NPPB or SITS,^8,11,12,14,33^ or even K^+^ channel blockers like quinine.^8^ Together, these data suggest that DIDS might have another target in cells besides Cl^−^ transporters and this might explain why DIDS is a generalized inhibitor of apoptosis.

It has been reported that dissolving DIDS in water produces reaction of its isothiocyanate reactive groups (-N=C=S) to form amino groups, which in turn can react with intact DIDS to form multimers that become a large impermeable multianion molecule due to the accumulation of sulfonate groups (–SO_3_^−^) in the multimer.^34^ Importantly, our stock solution of DIDS was dissolved in dehydrated DMSO. We think that this situation should decrease the probability of multimer formation. Accordingly, cells were incubated with monomeric, permeable and reactive DIDS. In this regard, we observed that cells incubated with DIDS and washed twice (to eliminate the unbound DIDS before the incubation with Ca^2+^ and PMP indicators) had reduced histamine-induced responses in both [Ca^2+^]_i_ and PMP hyperpolarization. These data indicate that DIDS modifies irreversibly cellular responses. Moreover, cell fluorescence due to the presence of DIDS was increased in these washed cells concentration dependently, arguing for DIDS incorporation into cells, because the packing of all this DIDS in the plasma membrane should not fluoresce due to internal quenching. Indeed, we were able to image DIDS inside cells and most likely attached to internal proteins.

Another target of DIDS is VDAC, a voltage-dependent anion channel that participates in cytochrome c release induced by different apoptotic stimuli.^17,25,35^ In this regard, DIDS inhibits mitochondrial membrane depolarization triggered by different inducers of apoptosis; this inhibition involves a mechanism that depends on VDAC.^8,12,16^ These data imply that DIDS reaches the interior of cells to interact with VDAC in the outer mitochondrial membrane to inhibit both cytochrome c release and mitochondrial membrane depolarization. Accordingly, cytochrome c release triggered by different type of apoptosis inducers, and in different cell types was strongly inhibited by DIDS.^5,16,25,35^ However, this effect of DIDS on cytochrome c release was not always mimicked by other Cl^−^ channel inhibitors, such as NPPB^12,14^ indicating that only DIDS may have an effect on VDAC. Importantly, our data have shown that STS-induced cytochrome c release in HeLa cells that had been in the absence of serum for 24 h cannot be blocked by DIDS, suggesting that in this case cytochrome c release does not depend on VDAC (not more than 20%).

Certainly, there are reports showing an inhibitory effect of DIDS in apoptosis that cannot be explained by DIDS blocking cytochrome c release;^11,36^ which is also the case shown here. This implies that DIDS is inhibiting apoptosis by a mechanism downstream to cytochrome c release.^7^ A currently overlooked action of DIDS in the field of apoptosis is its well-described activity as an alkylating agent, where isothiocyanate groups have a key part in this effect. Isothiocyanate groups can react with both amino and thiol groups,^21^ the latter are the reactive groups in the active site of all caspases. Since DIDS contains two reactive isothiocyanate groups, we think that one of these groups is reacting with the thiol group inactivating the protease, while the other might keep DIDS close to the active site by reacting with lysines nearby. In this regard, Lys 53, 57 and 210 are located close to the active site according to the human caspase-3 crystal structure.^37^ This scenario is supported by the observation that DIDS completely inhibited all caspases examined (capase-3, caspase-8 and caspase-9), in cell lysates containing previously activated caspases. However, cells incubated with DIDS displayed a complex pattern of inhibition, particularly for caspase-3. In short, 50 *μ*M DIDS inhibited 85% of STS-induced caspase-3 activity in cells. This means that there is 15% caspase-3 activity resistant to DIDS and this situation was not concentration dependent since 500 *μ*M DIDS did not change this percentage, although noise was reduced and the difference became significant. Caspase-3 resistant to DIDS might be the one located in the nucleus, where the presence of histones, proteins rich in lysine, would quench the alkylating effect of DIDS on caspase-3. This type of scenario might explain this observation because we could not find any caspase-3 activity resistant to DIDS in the cell-free extract. Importantly, this small fraction of caspase-3 resistant to DIDS was not observed in western blot, and we think that the explanation is that we used only supernatants to assess the cleavage of procaspase-3. Interestingly, it has been shown that DIDS at 500 *μ*M fully inhibited STS-induced apoptosis, while it had no effect on etoposide-induced apoptosis in Jurkat cells.^14^ It appears also that the mitochondrial transition pore was not involved; because cyclosporine A did not have any effect on the apoptosis triggered by either of the two inducers.^14^ Whether these data could be explained by etoposide activating a DIDS-resistant caspase-3 in Jurkat cells, might be interesting to look into. Moreover, it was completely unexpected to find that DIDS at high concentrations (500 *μ*M) induced a DEVDase activity that cannot be ascribed to caspase-3, since this was not blocked by the caspase-3 inhibitor. We did not study this activity any further, so we do not have any indication regarding the nature of this protease activated by DIDS. Interestingly, it has recently been reported that DIDS induces apoptosis in neurons as a consequence of increasing the expression of caspase-3.^38^ It is plausible that the ability of DIDS to stimulate a new DVEDase activity might explain why DIDS is not always protective either with long incubation times or at high concentrations.

In summary, DIDS besides the inhibition of anion channels both at the plasma membrane and in the mitochondria that might suffice to explain its inhibitory action in apoptosis can also inhibit the activity of caspases most likely by alkylating the reactive thiol group in the active site of these proteases. The latter might explain why DIDS is a generalized inhibitor of apoptosis when compared with other anion channel inhibitors. In fact, flufenamic acid did not have any effect on caspase-3 activity in cell-free extracts.

## Conclusion

Our work can be better explained based on previous observations that DIDS alkylates both thiol and amino groups in proteins and we propose that this is the reason for DIDS directly inhibiting caspase activity, in both intact and lysed cells. Our data suggest that 50 *μ*M DIDS is enough to inhibit STS-induced apoptosis and that higher concentrations (500 *μ*M) might have deleterious effects, probably because it induces a new DEVDase activity that is resistant to the caspase-3 inhibitor. This newly discovered action of DIDS to directly inhibit caspases, together with the other well-established actions of DIDS, such as inhibition of Cl^−^ fluxes and cytochrome c release via VDAC, might explain why DIDS is able to block staurosporine-induced apoptosis much better than other ion channel inhibitors.

## Materials and Methods

### Materials

Cell culture reagents were obtained from Gibco-Invitrogen (Grand Island, NY, USA). DIDS, staurosporine (STS), histamine, 2-Aminoethyl diphenylborinate (2-APB), flufenamic acid, gramicidin (Gram), Ac-DEVD-AMC (caspase-3 substrate), Ac-DEVD-CHO (caspase-3 inhibitor), Ac-LEHD-AFC (caspase-9 substrate), Ac-IETD-CHO (caspase-8 inhibitor), DMSO, Hoechst 33258 and protease inhibitor cocktail were purchased from Sigma-Aldrich (St. Louis, MO, USA). Ac-IETD-AMC (caspase-8 substrate) was obtained from Enzo Life Sciences (Lausen, Switzerland). Fura2-AM and DiBaC_4_ (3) (Bisoxonol) were obtained from Invitrogen (Molecular Probes, Eugene, OR, USA). z-VAD-fmk was purchased from Promega (Madison, WI, USA). Mouse anti-cytochrome c monoclonal antibody (Clone 7H8.2C12) and mouse anti-human PARP (Clone 4C10-5) were purchased from BD-Pharmingen (Chicago, IL, USA). Mouse anti-Caspase-3 (Clone 3G2) was obtained from Cell Signaling (Danvers, MA, USA). Mouse anti-*β*-tubulin was acquired from ZYMED (Carlsbad, CA, USA). Importantly, DIDS was prepared as a 50-mM stock in *dehydrated* DMSO, to avoid the hydrolysis of isothiocyanate groups.^34^ In control studies, 1% dehydrated DMSO neither induced caspase-3 activity nor blocked STS-induced caspase-3 activity (Supplementary Figure S6).

### Cell culture

HeLa cells were cultured in Dulbecco’s modified Eagle’s medium with high D-glucose (4.5 g/L), L-glutamine and sodium pyruvate (110 mg/L) and supplemented with both 5% fetal bovine serum and 3% newborn calf serum and with the antibiotic 1% penicillin/streptomycin. Cells were maintained at 37 °C, 5% CO_2_ and constant humidity. Serum was removed from the cells 24 h before commencing any experiment. These cells were then either induced to undergo apoptosis with staurosporine (STS), they were used to monitor changes in the plasma membrane potential (PMP) and [Ca^2+^]_i_, or they were incubated with DIDS to determine cell-associated DIDS fluorescence.

### Simultaneous determination of PMP and [Ca^2+^]_i_

After 24 h without serum, confluent HeLa cells were incubated with DIDS (50 or 500 *μ*M) or without for 2 h, then the cells were harvested, washed to remove unbound DIDS and the cell pellet was resuspended in saline solution containing (in mM): 121 NaCl, 5.4 KCl, 0.8 MgCl_2_, 1.8 CaCl_2_, 6 NaHCO_3_ and 25 Hepes, adjusted to pH 7.3 with NaOH^39^ at a cell density of 3×10^6^ cells/ml. Cell viability was always ⩾95% (0.08% Trypan Blue exclusion). The cell suspension was incubated with the Ca^2+^ indicator Fura-2AM (1 *μ*M per million cells) for 1 h. Thereafter, 250 *μ*l of the cell suspension (~7.5×10^5^ cells) was centrifuged to remove the extracellular Fura-2. The cells were resuspended in a cuvette with 2.5 ml of saline solution which contained 125 nM bisoxonol (PMP indicator). Dye loaded cells were sequentially excited at 340, 360 and 380 nm (for Fura-2) and 480 nm (for bisoxonol) and the emitted fluorescence signal collected at 530 nm using a QM-8 spectrofluorometer (PTI, Edison, NJ, USA). Once the bisoxonol fluorescence had leveled off, this signal was calibrated in mV by increasing the K^+^ conductance with histamine (100 *μ*M) followed by five consecutive additions of KCl (25 mM each time) and finally gramicidin (1 *μ*M) to fully depolarized the cells, as previously described.^25^ However, since there is no certainty that histamine produced complete hyperpolarization in those cells incubated with DIDS, ΔF/Fo data were not converted to mV. The Fura-2 signal was calibrated by applying digitonin (112 *μ*M) to obtain *R*_max_, followed by EGTA (10 mM) to obtain *R*_min_ and finally MnSO_4_ addition (9 mM) to obtain a measure of the autofluorescence. Fluorescence signals were acquired and analyzed with Felix 32 software (PTI).

### STS induction of caspase activity

HeLa cells were cultured in 6-well plates in the absence of serum for 24 h to enhance caspase-3 activation by STS (Supplementary Figure S7). To study the effect of DIDS, HeLa cells without serum for 19.5 h were preincubated with DIDS (50 or 500 *μ*M) for 30 min followed by the addition of STS (1 *μ*M) for an additional 4 h at 37 °C. To assess caspase activity, the culture medium was removed and added 500 *μ*l caspase lysis buffer to each well (50 mM Hepes, 5 mM DTT and 1% Triton X-100) and the plate was kept on ice with gentle shaking for 30 min before collecting the lysates.

### Determination of caspase activity

#### Determination of caspase-3 activity

Assays were carried out according to the specifications in the kit with slight modifications as previously described.^26^ Briefly, caspase-3 assay buffer contained in mM: 20 Hepes, 5 DTT, 2 EDTA and 0.1% Triton X-100, pH 7.4. For each one of the determinations, the assay buffer was supplemented with 50 *μ*L of the cell lysate and 1 *μ*M Ac-DEVD-AMC (caspase-3 substrate) with or without 2 *μ*M Ac-DEVD-CHO (caspase-3 inhibitor). These mixes were incubated at room temperature for 90 min. Caspase-3 activity was determined by subtracting the fluorescence signal obtained with the combination of substrate and inhibitor from the one with only substrate. The corrected signal obtained from cells incubated with STS was taken as 100% response. Figure 2 shows the fluorescence signal with substrate alone (closed columns) and the one obtained with the combination of substrate plus inhibitor (open columns) to demonstrate that 500 *μ*M DIDS induced a new DEVDase activity that was not blocked by caspase-3 inhibitor.

#### Determination of caspase-9 activity

The assay was carried out as previously described^40^ with slight modification. Caspase-9 assay buffer contained in mM: 100 MOPS, 10 DTT, 0.5 EDTA, 0.1% Triton X-100, pH 6.5 and 10% glycerol. The assay buffer with 100 *μ*l of the cell lysate was preincubated for 30 min at 32 °C before supplementing it with 1 *μ*M Ac-LEHD-AFC (caspase-9 substrate), either in the presence or in the absence of 2 *μ*M Ac-LEHD-CHO (caspase-9 inhibitor) and further incubated at 32 °C for 24 h. The fluorescence signal was analyzed as for the caspase-3 assay.

#### Determination of caspase-8 activity

The assay was carried according to the kit instructions with slight modification. Caspase-8 assay buffer contained in mM: 20 Hepes, 5 DTT, EDTA, 5% Sucrose and 0.1% Triton X-100 at pH 7.4. The assay buffer was supplemented with 1 *μ*M Ac-IETD-AMC (caspase-8 substrate) with or without 2 *μ*M Ac-IETD-CHO (caspase-8 inhibitor) and 50 *μ*l of cell lysate. This mix was incubated at 32 °C for 24 h. The fluorescence signal was analyzed as for the caspase-3 assay.

### Determination of the effect of DIDS on cell lysates

We obtained cell lysates using caspase lysis buffer from HeLa cells that had been previously incubated with STS for 4 h, a time selected because it induces maximal activity of caspases-3, -9 and -8. To test the direct effect of DIDS on caspase activity, we have previously activated caspases with STS and made cell lysates as described in the section of STS-induction of caspase activity. DIDS (50 *μ*M) was added to caspase activity assays and incubated at 37 °C for 45 min, this was followed by the addition of the corresponding caspase substrate to assess caspase activity as previously described.

### Immunodetection of cytochrome c release, PARP fragmentation and procaspase-3 processing

Cells cultured in 6-well plates were treated as previously described in the caspase activity section (24 h without serum, followed by treatment with STS alone or in the presence of DIDS). The cells were then washed with ice-cold PBS supplemented with the protease inhibitor cocktail and harvested with a cell lifter. The cell suspension was mechanically lysed by vortexing at top speed until the sample looked foamy (~30 s). Lysates were then centrifuged at 10^4^×g for 30 min to separate the supernatant (cytosolic fraction) from the pellet (particulate fraction). Thereafter, supernatant protein either 20 *μ*g (for cytochrome c and PARP) or 70 *μ*g (for procaspase-3) was separated by SDS-PAGE in a discontinuous gradient gel (5–20%, cytochrome c, PARP) or a continuous gel (16%, caspase-3), blotted in PVDF membranes (0.2 *μ*m) and revealed with anti-cytochrome c (1 : 1000), anti-PARP (1 : 1000) or anti-caspase-3 (1 : 1000). Anti-*β* tubulin (1 : 5000) was used as a loading control. We have verified the specificity of the antibodies used with Precision Plus Protein WesternC Standards from Bio-Rad cat 161-0376 (Hercules, CA, USA) as shown in Supplementary Figure S8. Importantly, we did not find any difference in the tubulin level among control cells and those incubated with DIDS with or without STS. In the case of cytochrome c, the optical density for each band was divided by the corresponding optical density of *β*-tubulin and this ratio obtained for extracts from cells incubated with STS was taken as 100%.

### Fluorescence determination of DIDS irreversibly bound to cells

To determine the fluorescence properties of DIDS in aqueous solution we put different concentrations (from 50 to 500 *μ*M) of DIDS in a QM-8 spectrofluorometer at 345 nm *λ*_ex_ and 420 nm *λ*_em_, as previously described^32^ and corrected for background fluorescence of the saline solution.

To estimate the amount of DIDS irreversibly bound to HeLa cells, the cells were incubated with DIDS (5, 50 or 500 *μ*M) or without for 2 h at 37 °C, then washed with DIDS-free PBS and resuspended at a density of 3×10^6^ cells/ml. Approximately 7.5×10^5^ of these cells were resuspended in 2.5 ml of saline solution to measure DIDS fluorescence using the wavelengths indicated in the previous paragraph. HeLa cell autofluorescence was subtracted from fluorescence of DIDS-preincubated HeLa cells and this was compared with fluorescence of DIDS in saline solution to estimate the amount of DIDS irreversibly bound to cells.

### Imaging of HeLa cells with confocal microscopy

#### Apoptotic nuclei determination

HeLa cells were grown on coverslips for 24 h without serum followed by corresponding treatment and fixed for 5 min with Bouin’s solution diluted 1 : 1 with culture media. Coverslips were washed four times with ethanol 70% and then incubated with Hoechst 33258 (5 *μ*M) overnight. Cell nuclei were analyzed with confocal microscopy (Zeiss LSM 700, Oberkochen, Germany) using a ×63 objective with 405 nm argon laser.

#### Imaging irreversibly bound DIDS in HeLa cells

Cells were grown on coverslips in the absence of serum for 22 h followed by incubation with DIDS (at either 50 or 500 *μ*M) or without for 2 h at 37 °C. Next cells were washed to eliminate unbound DIDS and fixed with Bouin’s solution 1 : 1 for 5 min followed by four washes with ethanol at 70%. DIDS fluorescence imaging was carried out with a confocal microscope (Zeiss LSM 700) using a ×63 objective and a 405-nm argon laser.

### Statistical analyses

All data are presented as mean±S.E.M., *n*= number of independent experiments. Statistical significance was evaluated by using ANOVA and Tukey multiple comparison tests. The difference between two means was considered to be significant if *P*<0.05.

## Abbreviations

2-APB: 2-amino ethoxydiphenyl borate
AVD: apoptotic volume decrease
DMSO: dimethyl sulfoxide
DTT: DL-dithiothreitol
EDTA: ethylenediaminetetraacetic acid
EGTA: ethylene glycol tetraacetic acid
PARP: poly ADP ribose polymerase
PMCA: plasma membrane calcium ATPase
PMP: plasma membrane potential
PVDF: polyvinyldene fluoride
STS: staurosporine
TNF: tumor necrosis factor
VDAC: voltage-dependent anion channel.
