# Supplementary material for: DIDS (4,4'-Diisothiocyanatostilbene-2,2'-disulfonate) directly inhibits caspase activity in HeLa cell lysates
Source: Cell Death Discov. 2015 Sep 28;1:15037–. doi: 10.1038/cddiscovery.2015.37 (PMC4979491; doi:10.1038/cddiscovery.2015.37)
Supplement: Supplementary Figure 1 [file cddiscovery201537-s1.pdf]

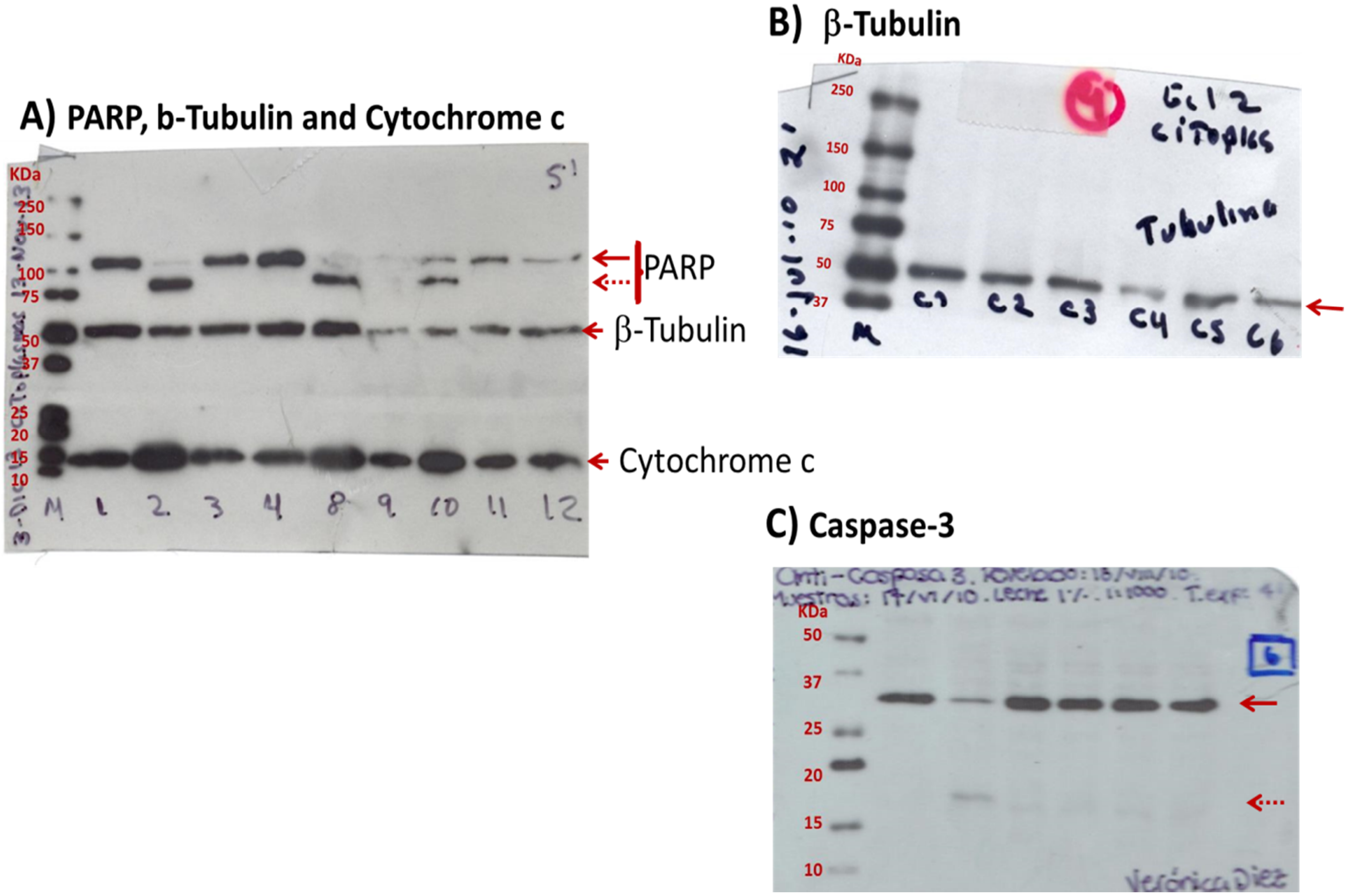

Figure S8. Western Blot images with protein standards. The specificity of the antibodies used in this work was verified with discontinuous-gradient polyacrylamide-gel electrophoresis, calibrated with Precision Plus Protein WesternC Standards from Bio-Rad cat 161-0376. Once we have determined the specificity of each one of the antibodies, we did not employ the MW standards anymore. A) WB for PARP (The antibody recognizes both complete (~113KDa) and fragmented PARP (~89 KDa, dotted arrow), β- tubulin (~50 KDa) and Cytochrome c (~15 KDa). B) WB for β-tubulin (~50KDa). C) WB for caspase-3 that recognizes both procaspase-3 (~35 KDa) and the cleaved active form of caspase-3 (~19 KDa, dotted arrow).
