# Supplementary material for: DIDS (4,4'-Diisothiocyanatostilbene-2,2'-disulfonate) directly inhibits caspase activity in HeLa cell lysates
Source: Cell Death Discov. 2015 Sep 28;1:15037–. doi: 10.1038/cddiscovery.2015.37 (PMC4979491; doi:10.1038/cddiscovery.2015.37)
Supplement: Supplementary Figure 2 [file cddiscovery201537-s2.pdf]

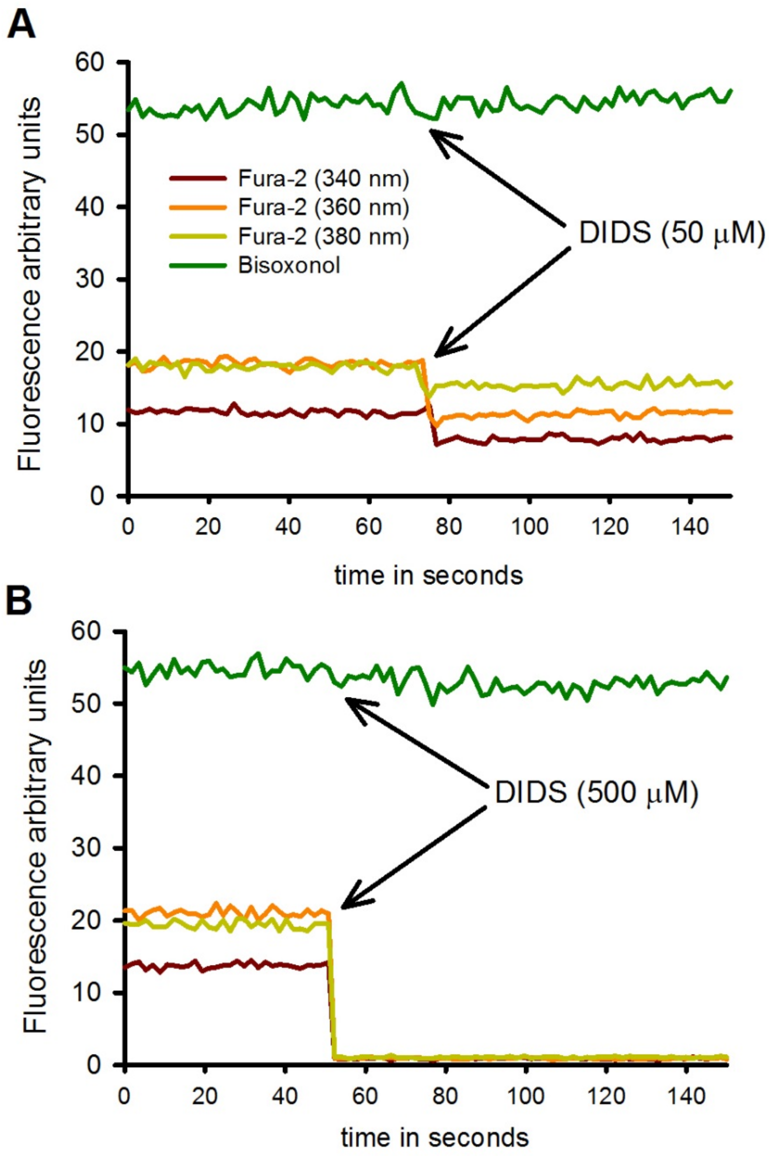

Figure S1. Effect of DIDS addition on fluorescence signals of HeLa cells loaded with both Fura-2 and Bisoxonol. HeLa cells were loaded with Fura-2 and Bisoxonol as indicated in the Methods section and fluorescence signal for both bisoxonol and three different excitation wavelengths for fura-2 were recorded before adding DIDS either 50 (panel A) or 500 μM (panel B) were indicated. The Fura-2 fluorescence signal was concentration dependently quenched at all wavelengths. However this was not the case for bisoxonol. Additionally, data suggest that DIDS does not modify plasma membrane potential in this recording conditions. In the case of Fura-2, this effect of DIDS cannot be ascribed to changes in the $[Ca^{2+}]_i$ because signal at 360nm was equally decreased. This quenching effect of DIDS on Fura-2 signal is the reason behind using washed cells that were preincubated with DIDS for recording Fura-2 and Bisoxonol fluorescence signals.
