# Supplementary material for: DIDS (4,4'-Diisothiocyanatostilbene-2,2'-disulfonate) directly inhibits caspase activity in HeLa cell lysates
Source: Cell Death Discov. 2015 Sep 28;1:15037–. doi: 10.1038/cddiscovery.2015.37 (PMC4979491; doi:10.1038/cddiscovery.2015.37)
Supplement: Supplementary Figure 3 [file cddiscovery201537-s3.pdf]

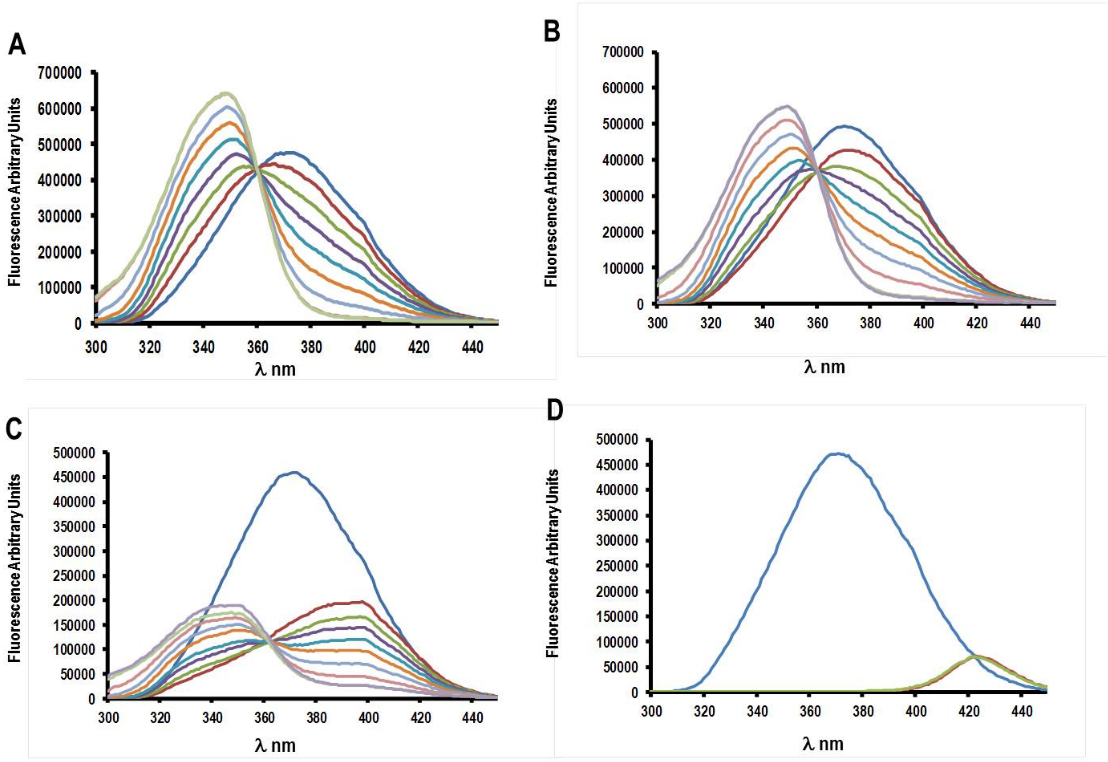

Figure S2. Effect of DIDS on excitation spectra of fura-2 dissolved in an aqueous solution and at different [Ca^{2+}]. Panel A shows the typical effect of increasing [Ca^{2+}] on fura-2 excitation spectra. Panel B, Fura-2 spectrum in the absence of Ca^{2+} (blue trace) was decreased by addition of 5 μM DIDS. However the application of Ca^{2+} still produced the characteristic shift in the excitation spectra of Fura-2 but at a rather smaller intensity. Panel C, Fura-2 spectrum in the absence of Ca^{2+} (blue trace) was strongly decreased by addition of 50 μM DIDS. Notice that there was still a shift in spectra in response to Ca^{2+} but intensity was strongly decreased and spectra highly distorted by DIDS. Panel D, Fura-2 spectrum in the absence of Ca^{2+} (blue trace) was totally quenched by addition of 500 μM DIDS.
