# Supplementary material for: DIDS (4,4'-Diisothiocyanatostilbene-2,2'-disulfonate) directly inhibits caspase activity in HeLa cell lysates
Source: Cell Death Discov. 2015 Sep 28;1:15037–. doi: 10.1038/cddiscovery.2015.37 (PMC4979491; doi:10.1038/cddiscovery.2015.37)
Supplement: Supplementary Figure 4 [file cddiscovery201537-s4.pdf]

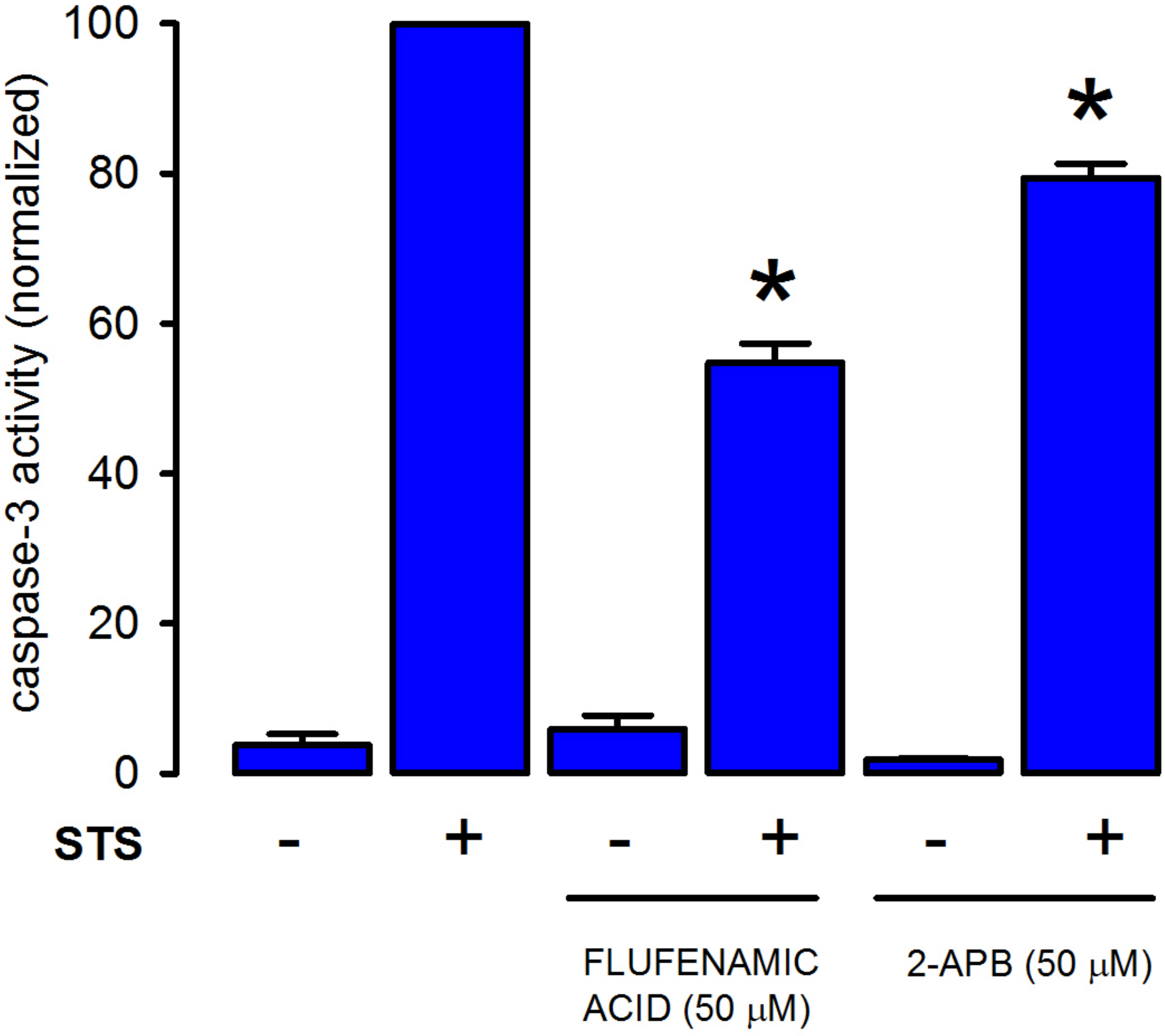

Figure S3. Different ion channel inhibitors reduce staurosporine-induced caspase-3 activation. HeLa cells that were in the absence of serum for 19.5 hours were preincubated for 30 min with either flufenamic acid or 2-APB followed by the addition of staurosporine and incubated for another 4 hours to then assess caspase-3 activity. Flufenamic acid, a known inhibitor of both cation and chloride channels, inhibited staurosporine-induced activation of caspase-3 by more than 40% while 2-APB, a non-specific inhibitor of Ca^{2+} permeable channels such as IP_3R, Orai and TRPs channels among others, by 20%. Data shown (n = 7) represents the mean ± SEM and * $p<0.5$ respect to staurosporine.
