# Supplementary material for: DIDS (4,4'-Diisothiocyanatostilbene-2,2'-disulfonate) directly inhibits caspase activity in HeLa cell lysates
Source: Cell Death Discov. 2015 Sep 28;1:15037–. doi: 10.1038/cddiscovery.2015.37 (PMC4979491; doi:10.1038/cddiscovery.2015.37)
Supplement: Supplementary Figure 5 [file cddiscovery201537-s5.pdf]

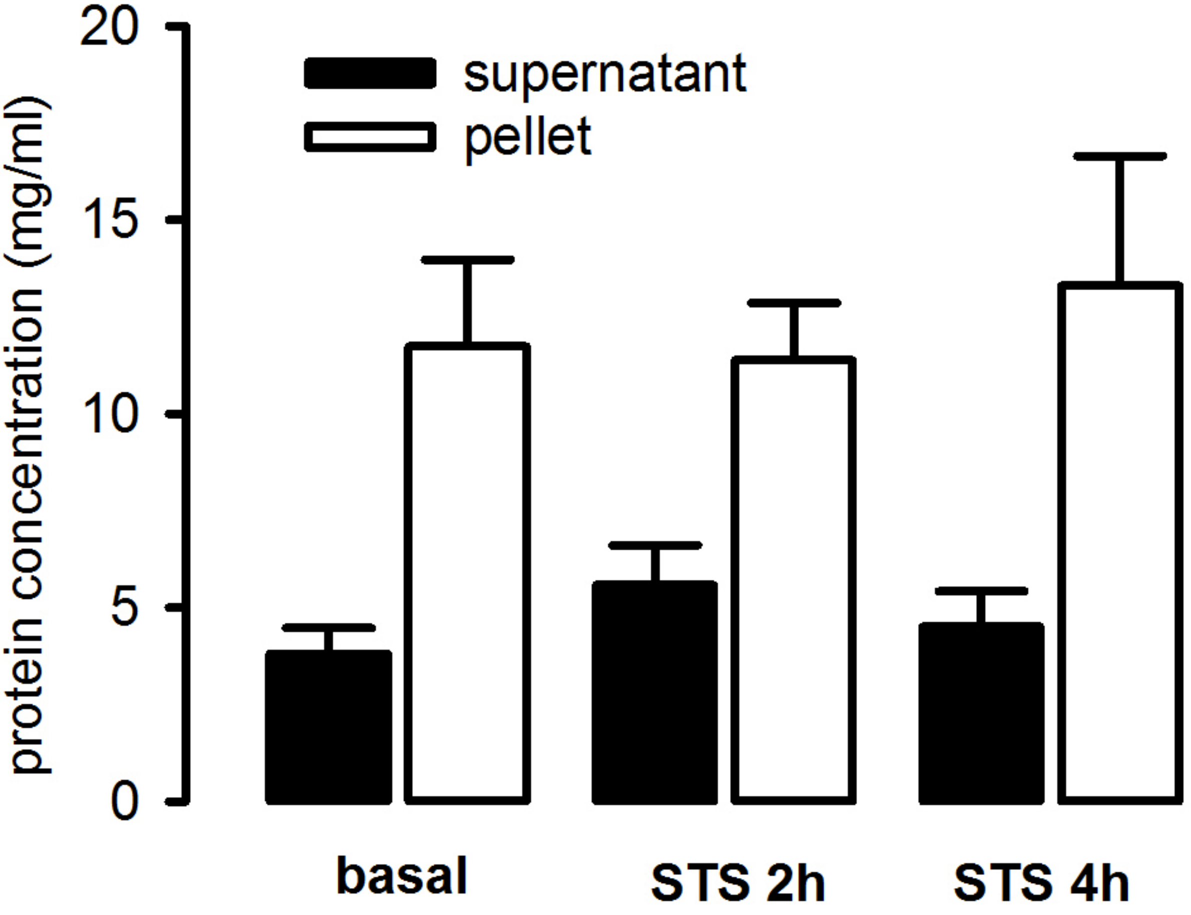

Figure S4. Protein concentration of HeLa cell lysates after being centrifuged at 10,000 x g. HeLa cells that had been in the absence of serum for 24 hours were mechanically lysed (using a vortex) and pellet and supernatant obtained by centrifugation at 10,000 x g. Protein levels are shown for both supernatant (filled bars) and pellet (open bars). Most of the protein of cell lysates was recovered in pellet and this difference was not affected by previous incubation of cells with Staurosporine (1 μM) for either 2 or 4 hours. All Western blots reported here used protein from supernatants and tubulin was used as a loading control, which did not show any difference by incubation with Staurosporine.
