# Supplementary material for: DIDS (4,4'-Diisothiocyanatostilbene-2,2'-disulfonate) directly inhibits caspase activity in HeLa cell lysates
Source: Cell Death Discov. 2015 Sep 28;1:15037–. doi: 10.1038/cddiscovery.2015.37 (PMC4979491; doi:10.1038/cddiscovery.2015.37)
Supplement: Supplementary Figure 6 [file cddiscovery201537-s6.pdf]

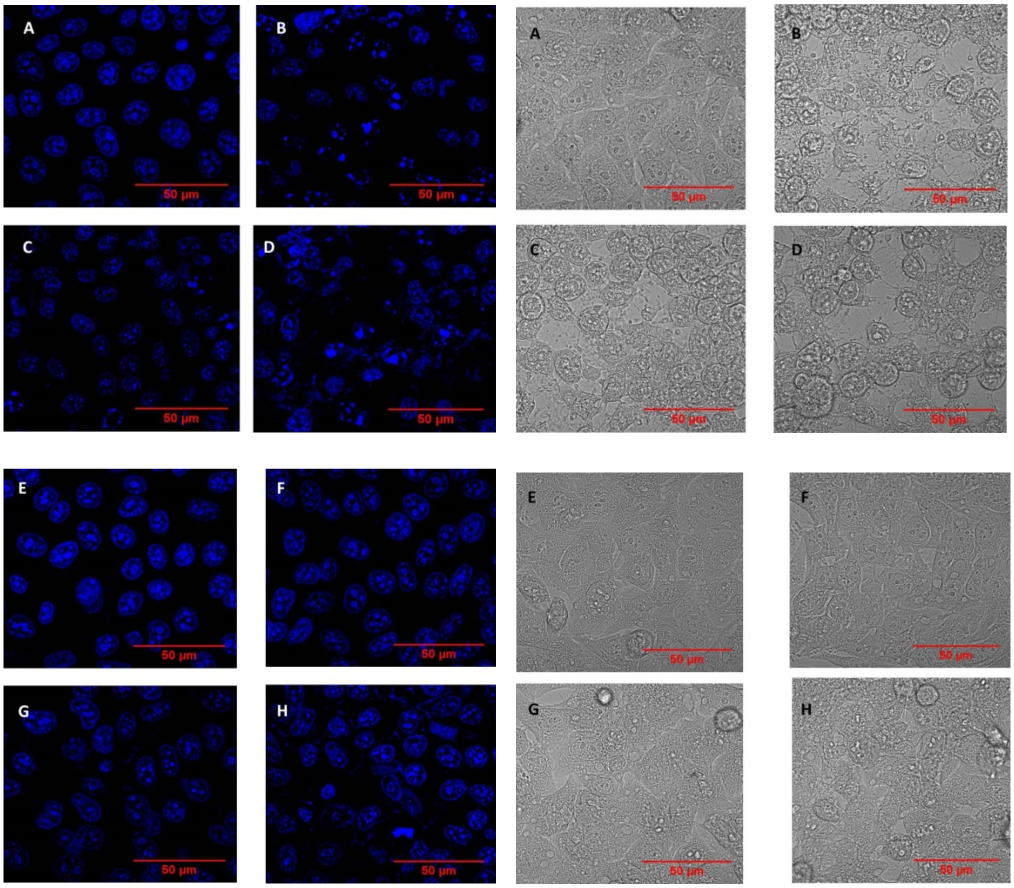

Figure S5. HeLa cell nuclei stained with Hoechst 33258 and visualized by confocal microscopy. HeLa cells that had been without serum for 24 hours were fixed with 1:1 Bouin´s solution for 5 min followed by 4 washes with ethanol 70% and incubated with Hoescht 33258 (5 µM) overnight. Panels on the left hand side show nuclei stained by Hoescht 33258 while panels on the right hand side show the corresponding bright field image. Panels A, control cells. Panels B, cells were incubated with staurosporine (1 µM) for 4 hours. Panels C, cells were preincubated for 30 min with DIDS (50 µM) and then followed by staurosporine for another 4 hours. Panels D, cells were preincubated for 30 min with DIDS (500 µM) and then with staurosporine for 4 hours. Panels E, cells incubated with 0.1% DMSO for 4 hours. Panels F, cells incubated with 1% DMSO for 4 hours. Panels G, cells incubated with DIDS 50 µM for 4 hours. Panels H, cells incubated with DIDS (500 µM) for 4 hours. These are sample images used to determine the percentage of apoptotic nuclei shown in Figure 5C.
