# Supplementary material for: DIDS (4,4'-Diisothiocyanatostilbene-2,2'-disulfonate) directly inhibits caspase activity in HeLa cell lysates
Source: Cell Death Discov. 2015 Sep 28;1:15037–. doi: 10.1038/cddiscovery.2015.37 (PMC4979491; doi:10.1038/cddiscovery.2015.37)
Supplement: Supplementary Figure 7 [file cddiscovery201537-s7.pdf]

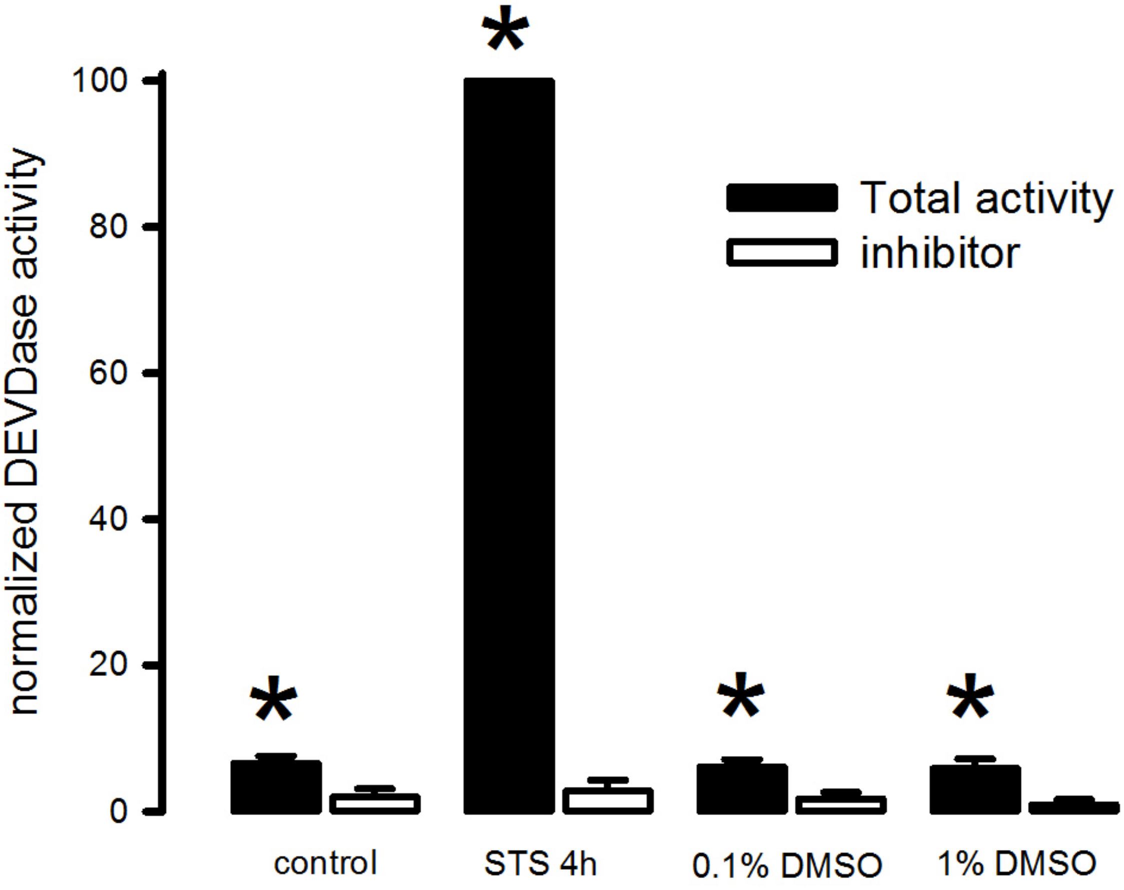

Figure S6. Effect of DMSO (vehicle) on Caspase-3 activity. HeLa cells that had been without serum for 20 hours were incubated for another 4 hours without serum and with either STS (1 μM) or 0.1% DMSO or 1% DMSO and caspase-3 activity was assessed as indicated in Methods section. Filled Bars show total DEVDase activity while open bars indicate the effect of Ac-DEVD-CHO, the caspase-3 inhibitor, on the DEVDase activity. Notice that this inhibitor eliminated all staurosporine-induced DEVDase activity. These data also show that 4-hr incubation with DMSO did not elicit any DEVDase activity in HeLa cells. (n = 5, * $p < 0.05$ when compared with inhibitor)
