# Supplementary material for: DIDS (4,4'-Diisothiocyanatostilbene-2,2'-disulfonate) directly inhibits caspase activity in HeLa cell lysates
Source: Cell Death Discov. 2015 Sep 28;1:15037–. doi: 10.1038/cddiscovery.2015.37 (PMC4979491; doi:10.1038/cddiscovery.2015.37)
Supplement: Supplementary Figure 8 [file cddiscovery201537-s8.pdf]

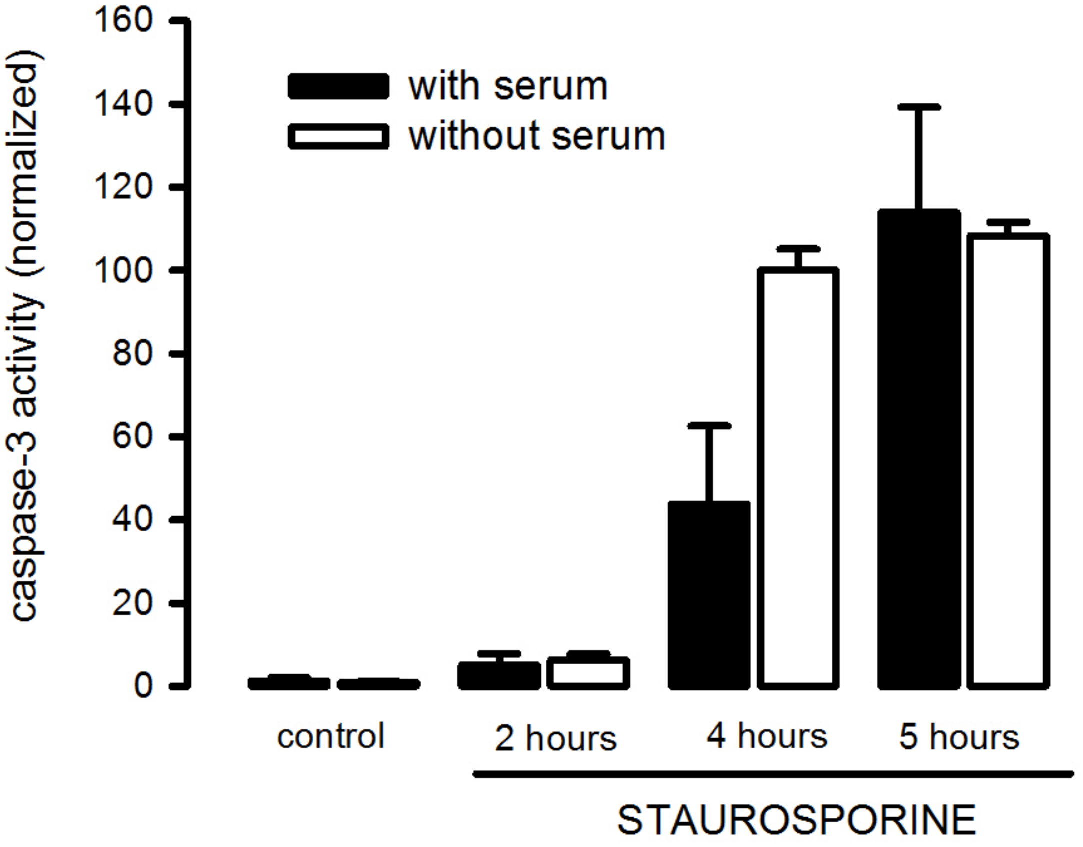

Figure S7. The absence of serum accelerates and facilitates the activation of caspase-3 by staurosporine in HeLa cells. HeLa cells were cultured with (closed bars) or without (open bars) serum for a total of 24 hours. Cells were preincubated with staurosporine (1 µM) for either 2, 4 or 5 hours before completing the 24-hr period of incubation. Caspase-3 activity was assessed as indicated in Methods section. Caspase-3 activity (total minus inhibitor) was not stimulated by 2-hr incubation period with staurosporine, but 4-hr period clearly triggered this activity in the absence of serum; also in the presence of serum, although with a large variability (closed bars). At 5-hr incubation period both conditions showed maximal activity although the variability in the presence of serum was still evident. These data suggest that staurosporine induces caspase-3 activity more consistently in the absence of serum. Longer incubation times did not increase any further caspase-3 activity (not shown).
